# Advances in the study of Müller glia reprogramming in mammals

**DOI:** 10.3389/fncel.2023.1305896

**Published:** 2023-12-14

**Authors:** Yi-Ming Guo, Xinyi Jiang, Jie Min, Juan Huang, Xiu-Feng Huang, Lu Ye

**Affiliations:** ^1^Shaanxi Eye Hospital, Xi’an People’s Hospital (Xi’an Fourth Hospital), Affiliated People’s Hospital of Northwest University, Xi’an, China; ^2^Department of Neonatology, The Second Affiliated Hospital and Yuying Children’s Hospital of Wenzhou Medical University, Wenzhou, Zhejiang, China; ^3^Zhejiang Provincial Clinical Research Center for Pediatric Disease, The Second Affiliated Hospital and Yuying Children’s Hospital of Wenzhou Medical University, Wenzhou, Zhejiang, China

**Keywords:** Müller cell, mammals, retinal regeneration, neural regeneration, reprogramming

## Abstract

Müller cells play an integral role in the development, maintenance, and photopic signal transmission of the retina. While lower vertebrate Müller cells can differentiate into various types of retinal neurons to support retinal repair following damage, there is limited neurogenic potential of mammalian Müller cells. Therefore, it is of great interest to harness the neurogenic potential of mammalian Müller cells to achieve self-repair of the retina. While multiple studies have endeavored to induce neuronal differentiation and proliferation of mammalian Müller cells under defined conditions, the efficiency and feasibility of these methods often fall short, rendering them inadequate for the requisites of retinal repair. As the mechanisms and methodologies of Müller cell reprogramming have been extensively explored, a summary of the reprogramming process of unlocking the neurogenic potential of Müller cells can provide insight into Müller cell fate development and facilitate their therapeutic use in retinal repair. In this review, we comprehensively summarize the progress in reprogramming mammalian Müller cells and discuss strategies for optimizing methods and enhancing efficiency based on the mechanisms of fate regulation.

## Introduction

Retinal diseases, whether triggered by traumatic incidents or endogenous factors, have the potential to inflict neuronal damage and apoptosis, ultimately culminating in irreversible blindness (Jonas et al., 2014; Fleckenstein et al., 2021). While therapeutic interventions such as photosensitization therapy (Busskamp et al., 2012), cell transplantation (Jin et al., 2019), and gene therapy (De Silva et al., 2017; DiCarlo et al., 2018) have been developed to address retinal degeneration and visual impairment, their success in *in vitro* and *in vivo* trials has been limited. Notably, gene therapy targeting the RPE65 gene variant in Leber’s congenital amaurosis has progressed to clinical trials (Weleber et al., 2016) and has demonstrated both safety and efficacy (Russell et al., 2017). However, these approaches often necessitate the introduction of exogenous genes or cells, as well as invasive surgical procedures, which carry the risk of physical damage and tumorigenicity (Cui et al., 2013; Feng et al., 2021). Consequently, numerous studies have been conducted to explore the potential of intraocular cell regeneration and the promotion of endogenous self-repair mechanisms in the retina.

Lower vertebrates, such as teleost fish and salamanders, have demonstrated the remarkable ability to self-repair retinal nerve damage, with Müller cells differentiating to fill the damaged areas and uphold retinal integrity (Raymond et al., 1988). However, in mammals, the response of Müller cells to retinal damage is limited to glia cell proliferation, prompting debates on their neurogenic potential and therapeutic applicability (Fischer, 2005; Bringmann et al., 2009). Consequently, investigations into the differentiation capabilities and fate regulation mechanisms of Müller cells have spurred the development of reprogramming techniques (Gao et al., 2021). These techniques leverage transcription factors, signaling pathways, and epigenetics to induce the targeted differentiation of restricted Müller cells (Goldman, 2014). Beyond exploiting the differentiation potential of Müller cells in mammals and directing their specific differentiation, these techniques offer novel tools to elucidate the developmental mechanisms of Müller cells and other retinal cells (Ooto et al., 2004; Sanhueza Salas et al., 2021). In summary, reprogramming presents a promising avenue for endogenous repair of the mammalian retina, ushering in new prospects for future research and therapeutic interventions.

In this review, we delve into the current understanding of the neurogenic potential of Müller cells in mammals and discuss diverse reprogramming strategies that can overcome their inherent limitations and induce their differentiation. We explore techniques to enhance reprogramming efficiency, counteract age-related declines in cell potential, and address discrepancies between *in vitro* and *in vivo* experiments, as well as variations across species ranging from chicken and fish to mouse and human. The research on Müller cell reprogramming provides valuable insights into the regulation of these cells’ fate and marks a notable advancement in the field of Müller cell-based retinal repair therapy, potentially opening doors for *in vivo* applications.

## The anatomy, function, and neurogenic potential of Müller cells in the retina

The retina, a highly intricate and organized structure, consists of six fundamental neurons arranged in three nuclear layers: the outer nuclear layer (ONL), the inner nuclear layer (INL), and the ganglion cell layer (GCL) (Hoon et al., 2014). Lineage tracing studies have revealed that retinal neurons and Müller cells share a common origin as unipotent retinal neuroepithelial cells (Lenkowski and Raymond, 2014). Maintaining the structure and function of the retina heavily relies on the presence of endogenous Müller cells, which represent the predominant Müller cell type in the mammalian retina, constituting approximately 90% of all Müller cells. Müller cells possess a bipolar cytoarchitecture that spans the entire thickness of the retina and establish interactions with all retinal cell types (Bringmann et al., 2006).

**FIGURE 1:**
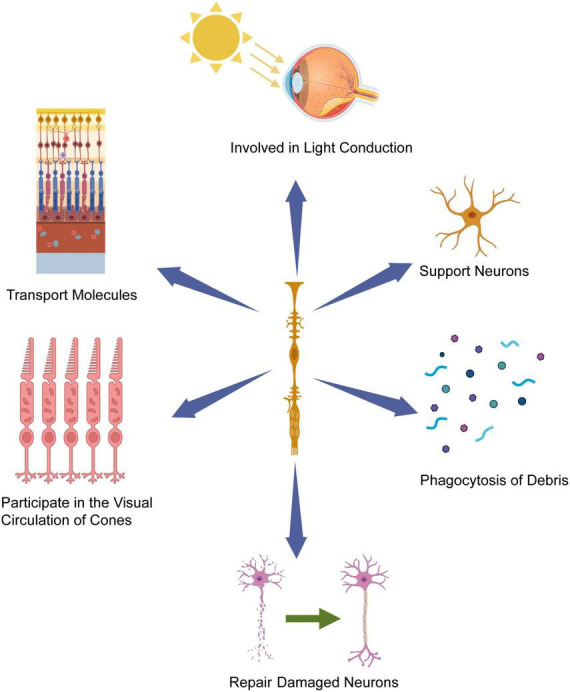
Müller cells’ contribution to retinal homeostasis and function. Müller cells possess cell bodies extending across the entire width of the retina, with their nuclei situated within the inner nuclear layer (INL). Leveraging their radial orientation and strategic placement, Müller glia cells play a pivotal role in offering crucial support and maintenance functions to the retinal tissue, including light conduction in retina, participating in the visual circulation of cones and repairing damaged neuron. Created with BioRender.com.

Müller cells play a crucial role in retinogenesis, primarily through their ability to transmit various molecules between different retinal cells (Too and Simunovic, 2021). Furthermore, they support neurons by releasing trophic factors, neurotransmitters, and regulating extracellular ion homeostasis. Additionally, Müller cells are actively engaged in the visual cycle specific to cone cells, as they phagocytose the outer segments of cone cells, promoting their metabolism and production. These Müller cells also participate in the phagocytosis of cellular debris and dynamically interact with microglia to regulate debris removal (Reichenbach and Bringmann, 2020). Notably, Müller cells can function as a phototransduction pathway, influencing retinal responses by transmitting light information (Goldman, 2014; Bejarano-Escobar et al., 2017). Collectively, Müller cells are strategically positioned to monitor and maintain intraretinal homeostasis, thus contributing to the establishment of retinal structure and function. Gaining a comprehensive understanding of the dynamic and intricate contributions of Müller cells to retinal homeostasis and function is vital in the pursuit of novel therapeutic approaches for the treatment of retinal diseases (Figure 1).

Müller cells have assumed a critical role in the regenerative response of damaged retinas in lower vertebrates, such as zebrafish, and exhibit robust regenerative potential by differentiating and replacing damaged retinal neurons to uphold retinal integrity (Bernardos et al., 2007; Nagashima et al., 2013). While mammalian Müller cells have limited differentiation capacity, they display reactive gliosis in the face of disease or injury, characterized by morphological changes, upregulation of various markers, dedifferentiation, and migration of nuclei to the apical surface (Bringmann et al., 2006, 2009).

Remarkably, the regenerative response of mammalian Müller cells bears striking resemblance to the early stages of regeneration observed in fish (Wan and Goldman, 2016) or chicken (Fischer and Reh, 2001) retinas and even exhibits similar characteristics to late retinal progenitors in their response (Jadhav et al., 2009). Some studies showed some of reactive Müller glia cells in the traumatic or ischemic brain acquire neurosphere-forming capacity including multipotency and long-term self-renewal *in vitro* and even share endogenous neural stem cells (NSCs) exhibit stem cell hallmarks largely *in vitro* (Götz et al., 2015). Single-cell assays have also revealed transcriptional similarities between Müller cells and retinal progenitor cells (Roesch et al., 2008), and neuronal production has been observed after Müller cell transplantation in rodents and humans (Das et al., 2006). Furthermore, the ability of Müller cells to exit quiescence and re-enter the cell cycle in the context of retinal injury relies heavily on factors such as species and severity of the injury. For example, in mice with photoreceptor damage, Müller cells express cyclin D1 but do not progress to the S phase of the cell cycle, implying that, in the context of mice, Müller cells face impediments in advancing into the S-phase of the cell cycle (Joly et al., 2011), whereas in a rabbit model of retinal detachment, an identifiable subset of cells labeled with anti-BrdU, likely representing Müller cells, seems to cease the expression of widely recognized Müller cell marker proteins, suggesting a potential dedifferentiation of some of these cells over time (Ooto et al., 2004; Lewis et al., 2010).

Overall, mammalian Müller cells may not exhibit the same reparative potential as retinal progenitors *in vivo*, their response to injury suggests a latent neurogenic potential, implying that Müller cells can be transformed into stem cells under appropriate conditions (Gao et al., 2021). And Müller cells may emerge as pivotal contributors to the regenerative response of damaged retinas, holding potential for future therapeutic applications.

The differentiation of mammalian Müller cells into specific cell types within the retina offers potential for neural repair, despite their tendency to inhibit regenerative processes through reactive gliosis following retinal damage. Recent studies have successfully demonstrated the neurogenic potential of Müller cells in retinal explants and mouse models of retinal degeneration, dispelling previous doubts regarding their regenerative capacity. Ooto et al. (2004) work confirmed that Müller cells can serve as a source of new neurons in a rat model of NMDA (N-methyl-d-aspartate)-induced excitotoxic retinal injury. Subsequent studies further supported the neurogenic potential of Müller cells in mouse models of retinal explants and retinal degeneration (Osakada et al., 2007). Additionally, primary human Müller cells have shown promise in generating optic rod cells (Jayaram et al., 2014) and ganglion cells (Singhal et al., 2012) through culture and transplantation into mouse models of retinal damage. Notably, human Müller glia cells exhibit accelerated differentiation toward optic rod cells compared to conventional pluripotent stem cells, emphasizing their advantage in neural repair (Giannelli et al., 2011).

External administration of growth factors such as EGF and FGF1 can also stimulate Müller cells proliferation in NMDA-injured retinas through various signaling pathways, including PI3K/AKT, MAPK/ERK1/2, and BMP/Smad1/5/8 (Karl et al., 2008; Ueki and Reh, 2013). Interestingly, even sub-toxic levels of glutamate can induce the re-entry of Müller cells into the cell cycle, indicating the involvement of complex signaling pathways in this process (Takeda et al., 2008). These findings hold great promise for the use of Müller cells in retinal repair therapy, highlighting their regenerative capacity and the potential for future therapeutic applications. Overall, these studies not only confirm the full but limited pluripotent potential of Müller cells in mammals and the feasibility of promoting and regulating their proliferation and differentiation through various interventions but also suggest their suitability for retinal repair therapy.

Therefore, harnessing the neurogenic potential of Müller cells, abundant, specialized, and potent within the retina, represents a significant milestone in advancing regenerative medicine in mammals. Nonetheless, it is crucial to carefully consider and explore the perspectives and approaches through which such cellular transformations can be achieved.

## Müller cell reprogramming in mammals

**FIGURE 2:**
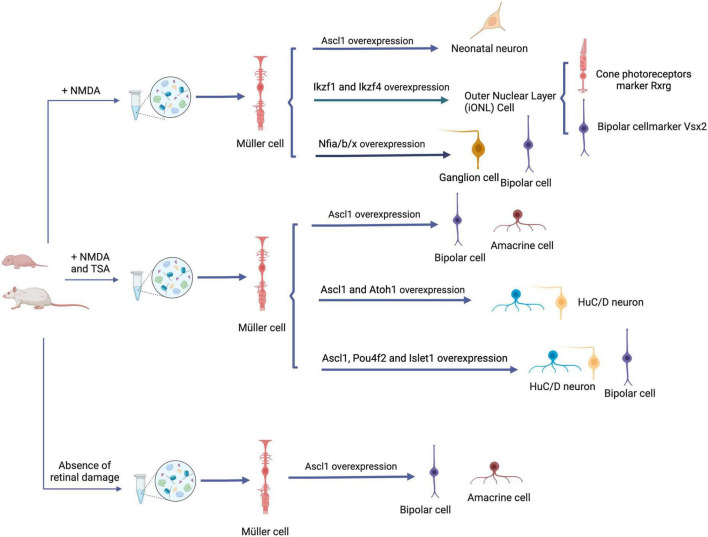
Reprogramming strategies for Müller cells in retinal repair. Overexpression of ASCL1 in juvenile mice following NMDA-induced retinal damage leads to Müller cell reprogramming, resulting in the generation of functional neonatal neurons. Simultaneously, overexpression of Ikzf1, Ikzf4, and Nfia/b/x facilitates the transition of retinal functional neurons, highlighting an alternative reprogramming strategy. Application of the HDAC inhibitor TSA help to induce Müller glia cell reprogramming after retinal injury in adult mice. This reprogramming process generates functional interneurons, such as amacrine and bipolar cells, and this can be realized by the overexpression of Ascl1 + Atoh1 or Ascl1 + Pou4f2 + Islet1. Even in the absence of retinal damage, Ascl1 overexpression is sufficient to realize the reprogramming. ASCL1, Achaete-scute homolog 1; NMDA, N-methyl-d-aspartate; HDAC, histone deacetylase; TSA, Histone deacetylase inhibitor trichostatin-A; Ikzf1 (a.k.a. Ikaros), the homolog of Drosophila hb; Pou4f2, P Pou4f2, a class IV, Pit-Oct-Unc (POU) homeodomain TF; Islet1, a LIN, Islet1, MEC3 (LIM) homeodomain TF; Atoh1, a proneural basic helix–loop–helix (bHLH) transcription factor. Created with BioRender.com.

Takahashi and Yamanaka (2006) made a significant breakthrough by successfully inducing mouse embryonic fibroblasts into iPSCs (induced pluripotent stem cells) *in vitro*, expanding and revolutionizing the field of cellular reprogramming. This remarkable transformation of cell identity, accomplished through the activation of specific transcription factors and related regulatory mechanisms, holds the potential to be applied to mammalian Müller cells. By utilizing reprogramming techniques, Müller cells could overcome their inherent limitations and acquire enhanced versatility in their applications (Table 1). The concept of reprogramming offers an exciting avenue to unlock the latent regenerative potential of Müller cells and further expand their therapeutic capabilities (Figure 2).

**TABLE 1:** Key molecules of Muller cell reprograming in mammals.

Key molecules	Animal model	Test	Result	References
Ascl1	Young mice +	*In vitro*	Retinal progenitors	Pollak et al., 2013
	Young mice +	*In vitro*	Amacrine cells, Bipolar cells and Photoreceptors	Ueki et al., 2015
	Adult mice + TSA	*In vitro*	Bipolar cells and amacrine cells	Jorstad et al., 2017
Ascl1 + Atoh1	Adult mice + TSA	*In vitro*	Retinal ganglion cells	Todd et al., 2021
Ascl1 + Atoh1	Adult mice (without retinal injury)	*In vitro*	Retinal ganglion cells and amacrine cells	Todd et al., 2021
Ascl1 + Pou4f2 + Islet1	Adult mice + TSA	*In vitro*	Retinal ganglion cells, amacrine cells and bipolar cells	Todd et al., 2022
PTBP1 (knockdown)	Adult mice	*In vivo*	Retinal ganglion cells	Zhou et al., 2020
	Adult mice	*In vivo*	No neuronal conversion	Hoang et al., 2022
	Adult mice	*In vivo*	No conversion to retinal ganglion cells	Xie et al., 2022
	Adult mice	*In vivo*	No neuronal conversion	Yang et al., 2023
β-catenin (Wnt-Lin28-let7 miRNA signaling)	Adult mice (without retinal injury)	*In vivo*	Amacrine cells	Yao et al., 2016
	Adult mice (without retinal injury)	*In vivo*	Rod photoreceptors	Yao et al., 2018
Ikzf1 + Ikzf4	Adult mice	*In vivo*	Cone photoreceptors and bipolar cells	Boudreau-Pinsonneault et al., 2023
Nfia/b/x	Adult mice	*In vivo*	Retinal bipolar cells and amacrine cells	Hoang et al., 2020

TSA, histone deacetylase inhibitor trichostatin-A.

### ASCL1-mediated reprogramming of Müller cells

Achaete-scute homolog 1 (ASCL1), a core transcription factor, has emerged as a key player in the transformation of Müller cells into retinal progenitors, as demonstrated in zebrafish studies (Elsaeidi et al., 2018). Interestingly, upregulation of ASCL1 has been observed in a subset of Müller cells in young mice following retinal damage, suggesting its potential involvement in regulating the fate of Müller cells in mammals (Loffler et al., 2015). However, no changes in ASCL1 expression were observed in the damaged retinas of adult mice treated with NMDA (Karl et al., 2008), indicating potential differences in the role of ASCL1 during retinal regeneration in different age groups.

Noteworthy advancements have been made in investigating the reprogramming potential of ASCL1 in Müller cells. Studies have shown that overexpression of ASCL1 can reprogram mouse Müller cells *in vitro*, leading to their re-entry into the cell cycle and upregulation of retinal progenitor cell-specific gene expression. Furthermore, these reprogrammed Müller cells differentiate into neurons exhibiting characteristic morphology and expressing retinal cell markers (Pollak et al., 2013). Ueki et al. (2015) successfully achieved Müller cell reprogramming in NMDA-injured retinas of mice through ASCL1 overexpression, with higher efficiency observed in young mice compared to adults. These findings suggest that the regenerative potential of Müller cells diminishes with age. However, in P16 mice with significantly reduced neurogenic potential, ASCL1 overexpression alone proved insufficient for Müller cell reprogramming.

Recent studies have taken an epigenetic approach to explore ASCL1-mediated reprogramming in Müller cells. It has been observed that the promoters of reprogramming-associated genes in mammalian Müller cells, such as ASCL1a, Lin28, and Hbegfa, are typically hypomethylated. Overexpression of ASCL1 has been shown to activate chromatin and enhance gene activity (Pollak et al., 2013), while the ASCL1-Wnt axis can regulate chromatin remodeling by influencing the expression of histone modifying enzymes during Müller cell reprogramming (Aldiri et al., 2013). Jorstad et al. (2017) discovered that combining ASCL1 overexpression with the HDAC (histone deacetylase) inhibitor TSA induced reprogramming of Müller glia cells in adult mice after retinal injury. This reprogramming resulted in the production of functional interneurons, including long-free synapses and bipolar cells (Jorstad et al., 2017). High-throughput sequencing further revealed that the HDAC inhibitor increased accessibility to critical gene loci in the chromatin of Müller cells, compensating for the reduced neurogenic potential associated with aging and improving reprogramming efficiency. These findings highlight the crucial role of ASCL1 in Müller cell reprogramming and provide insights into the epigenetic mechanisms underlying this process. Manipulating ASCL1 expression and utilizing epigenetic modifiers offer promising strategies for enhancing the regenerative capacity of Müller cells, particularly in the context of retinal repair therapy.

Noticeably this regenerative process is characterized by inefficiency. By testing additional transcription factors (TFs) for their ability to direct regeneration to particular types of retinal neurons, Todd’s research has revealed that the combination of Ascl1 and Atoh1 is remarkably effective in promoting neurogenesis, even in the absence of retinal injury. This combined approach not only boosts efficiency but also leads to the generation of a diverse array of retinal neuron types, with the majority exhibiting characteristics typical of retinal ganglion cells (Todd et al., 2021, 2022). The optimization of ASCL1 overexpression also can be achieved through synergistic combinations with other signaling pathways and microRNAs, resulting in enhanced reprogramming efficiency. Notably, Elsaeidi et al. (2018) demonstrated that the co-expression of ASCL1 and Lin28 significantly augmented the proliferative response in the late-stage mouse retina following NMDA treatment, indicating an improved reprogramming efficiency. Furthermore, differential expression of molecules in the JAK/STAT pathway during this reprogramming process suggests that the regulation of STAT signaling influences ASCL1-mediated reprogramming (Ueki et al., 2015; Jorstad et al., 2020). Inhibition of the Notch pathway has also been shown to induce ASCL1 expression in Müller cells, further supporting its involvement in the reprogramming process (Loffler et al., 2015). Moreover, the reprogramming effect of ASCL1 can be enhanced by the overexpression of miR-5, miR-124, and miR-9 (Wohl and Reh, 2016). In summary, the overexpression of ASCL1 is a pivotal strategy for reprogramming mammalian Müller glia cells, and its neurogenic potential can be further harnessed by synergistically combining it with other transcription factors, signaling pathways and microRNAs.

### PTBP1-mediated reprogramming of Müller cells

Polypyrimidine tract-binding protein 1 (PTBP1), a member of the PTB family, is ubiquitously expressed and serves as a classical post-transcriptional regulator of gene expression. It exerts control over mRNA production, translation, stabilization, and localization, playing a crucial role in neuronal cell growth and differentiation (Oberstrass et al., 2005). The activity of PTBP1 is modulated by binding sites on pre-mRNA, either activating or inhibiting its function (Corrionero and Valcarcel, 2009). For example, PTBP1 can repress the neural transcription program by regulating the expression of the transcription factor Pbx1 (Linares et al., 2015). Furthermore, PTBP1 interacts with the neural-specific long-strand non-coding RNA Pnky in neural stem cells, modulating the cleavage and expression of several core transcription factors associated with cell phenotype. Dual knockdown of PTBP1 and Pnky significantly reduces the number of neural stem cells, promoting neurogenesis and differentiation (Ramos et al., 2015). Consequently, PTBP1 plays a critical role in the development of neurogenic cells and holds great potential for applications in neuromodulation research.

Recently, Zhou et al. (2020) achieved targeted knockdown of PTBP1 using a specific CRISPR-CasRx system, enabling the *trans-*differentiation of Müller glia cells into retinal ganglion cells (RGCs) and anaplastic cells in both healthy and NMDA-injured adult mouse retinas. The reprogrammed ganglion cells projected correctly to the brain and exhibited a reparative role in a model of permanent visual damage (Zhou et al., 2020). This research highlighted the role of PTBP1 downregulation in orchestrating the reprogramming of Müller glial cells, thereby contributing to the partial restoration of visual function. And the process involves a variety of modifications and regulatory changes. For example, PTBP1-mediated identity transformation of Müller cells involves various epigenetic modifications, including the regulation of miR-9 and miR-124, which in turn can modulate PTBP1 activity (Hu et al., 2018). PTBP1 also competes for miRNA binding, resulting in changes in cell fate (Xue et al., 2013). Additionally, PTBP1-mediated reprogramming involves the repressor element 1-silencing transcription factor (REST), which not only orchestrates genetic modifications but also influences the expression of specific genes during the differentiation of neurogenic cells. Consequently, targeting the PTBP1/miRNA-124/REST axis holds significant potential to impact the altered fate of Müller cells upon PTBP1 knockdown (Gao et al., 2021).

Nevertheless, the reprogramming achieved through PTPB1 deletion remains a subject of controversy (Chen, 2021; Wang and Zhang, 2022, 2023; Hao et al., 2023; Hoang et al., 2023). Hoang’s research, employing a combination of genetic lineage tracing, single-cell RNA sequencing (scRNA-seq), and electroretinogram analysis, has demonstrated that the selective induction of either heterozygous or homozygous loss-of-function mutants of PTBP1 in adult retinal Müller glia does not result in any observable level of neuronal conversion (Hoang et al., 2022). Similarly, through fate-mapping experiments that allowed lineage tracing of Müller glia independently of the adeno-associated virus (AAV)-mediated labeling system, Xie’s research has demonstrated that Ptbp1 downregulation using CRISPR-CasRx or small hairpin RNA is insufficient to transform Müller glia into retinal ganglion cells (RGCs). This re-evaluation suggests that the initial conclusion of Müller glia-to-RGC conversion may be attributed to the unintended labeling of endogenous RGCs due to leakage in the labeling process (Xie et al., 2022).

Furthermore, in addressing the inquiry regarding the precision of studies asserting the efficacy of glia-to-neuron reprogramming within the retinal context, Lee employed GFAP mini promoter-driven adeno-associated virus (AAV) vectors. These vectors were designed to concurrently facilitate the overexpression of the mCherry reporter and the candidate transcription factors that were anticipated to trigger the transformation of glial cells into neurons. This was done in conjunction with the prospective genetic labeling of retinal Müller glia through inducible Cre-dependent GFP reporters. The ultimate outcome of this approach revealed substantial insert-dependent effects on AAV-based GFAP mini promoter specificity, rendering it unsuitable for deducing cell lineage relationships when investigating glia-to-neuron conversion in the retina (Le et al., 2022). Yang conducted a comparable study using Cas13X and labeled astrocytes by fusing an HA tag to Cas13X (Cas13X-NLS-HA) for Ptbp1 suppression. Interestingly, their findings revealed no conversion of astrocytes into neurons in the mouse striatum using the HA-tagged labeling system, in contrast to the results obtained from previous studies that used the GFAP-driven tdTomato labeling system (AAV-GFAP:tdTomato-WPRE) (Yang et al., 2023). Consequently, these discoveries not only cast significant doubt on the reprogramming effects associated with Ptbp1 knockdown but also underscore the critical importance of incorporating genetic manipulation and lineage-tracing techniques when examining cell-type conversions.

In conclusion, the assertion suggesting that PTBP1 knockdown can transform Müller glia into retinal ganglion cells has encountered significant scrutiny and skepticism. The veracity of this claim is under rigorous scrutiny, prompting a demand for further investigation and meticulous evaluation.

## Müller cell reprogramming mediated by other signaling pathways

The Wnt/β-catenin signaling pathway has been shown to confer neuroprotection through Müller cells in a mouse model of retinal degeneration (Patel et al., 2015). Conversely, the overexpression of β-catenin and microRNA Lin28 alone has been demonstrated to be sufficient for Müller cells to re-enter the cell cycle. Subsequent studies have revealed the interconnection between these two factors through the Wnt signaling pathway (Yao et al., 2016). Specifically, β-catenin activates the Wnt signaling pathway in the intact retina of adult mice, thereby promoting Müller cell proliferation. Downstream of the Wnt signaling pathway, Lin28 activates transcription, and upregulation of Lin28 can regulate the neurogenic potential of Müller cells through the let-7 miRNAs. The β-catenin/Wnt/Lin28 axis is, therefore, critical to Müller cell reprogramming. The effectiveness of transgenically introducing β-catenin to reactivate the Müller cell cycle and induce reprogramming was highlighted in Yao’s study. In this investigation, the endogenous reprogramming of photoreceptors from Müller cells was achieved using this approach in a mouse model of congenital night blindness, leading to a substantial enhancement in visual acuity (Yao et al., 2018). However, it is important to acknowledge that the methodology employed in this process has faced scrutiny for its technical aspects, particularly in the context of AAV-mediated reprogramming experiments. This scrutiny is underscored by some discussions (Martin and Poché, 2019; Blackshaw and Sanes, 2021) and further examination and consideration of these technical concerns may be warranted.

The role of the Notch pathway in Müller cell reprogramming is also noteworthy. To trigger a regenerative response from Müller glia, it is essential to surpass a specific injury-derived signal threshold, known as the Müller glia injury-response threshold (Fausett and Goldman, 2006). Certainly, Müller cell do not initiate a regenerative response when the level of cell death remains minimal (Iribarne et al., 2019; Lessieur et al., 2019). The broad inhibition of Notch signaling throughout the entire retina extends the region of injury-responsive Müller glia at the focal injury site. This observation implies that Notch signaling plays a regulatory role in modulating Müller glia’s injury-response threshold (Wan and Goldman, 2016). Sahu’s research specifically unveiled that Notch signaling contributed to an increase in chromatin accessibility and the expression of specific genes associated with regeneration in the uninjured retina. Within this context, two Notch effector genes, hey1 and id2b, emerged as crucial players, signifying a divergence in the Notch signaling pathway. Importantly, these genes differentially regulated both Müller glia’s injury-response threshold and the proliferation of Müller glia-derived progenitors (Sahu et al., 2021). Besides, in the context of zebrafish retinal regeneration, the control of Müller glia proliferation can be effectively mediated by Notch3 and DeltaB, exerting negative regulation (Campbell et al., 2021). In mammals, treatment of retinal explants with the Notch pathway ligand Jag1 has been found to promote Müller cell re-entry into the cell cycle (Del Debbio et al., 2010).

The inhibition of the Notch pathway has been demonstrated to enhance photoreceptor regeneration in models of progressive degeneration (zebrafish cep290 mutants), while concomitant immunosuppression has shown promise in preventing photoreceptor loss (Fogerty et al., 2022). Furthermore, Notch signaling can synergize with the Wnt pathway to facilitate the formation and proliferation of photoreceptors during Müller cell reprogramming. This process leads to an increase in the number of photoreceptor cells exhibiting a rod photoreceptor phenotype and responding to light in the S334ter rat model (Yao et al., 2016). Therefore, the Notch pathway represents a crucial component in the reprogramming of Müller cells, offering valuable insights into the development of therapeutic strategies for retinal repair.

Through the development of a conditional gene expression system, which enables the swift screening of potential reprogramming factors in murine retinal glial cells, combined with genetic lineage tracing, Cayouette’s research team has recently made a significant discovery. Their findings reveal that the co-expression of the early temporal identity transcription factors Ikzf1 and Ikzf4 is adequate for the direct conversion of Müller glial (MG) cells. These converted cells subsequently migrate to the outer nuclear layer (ONL), a location typically occupied by photoreceptor cells. Notably, in the absence of retinal injury, the *in vivo* expression of Ikzf1/4 within MG primarily yields iONL cells. These iONL cells exhibit molecular characteristics akin to bipolar cells, although a subset of them demonstrates positive staining for Rxrg, a marker associated with cone photoreceptors (Boudreau-Pinsonneault et al., 2023).

The reprogramming of Müller cells is influenced by the Hippo signaling pathway, with the Yes-associated protein (YAP) playing a critical role. Under normal conditions, YAP is suppressed through phosphorylation, inhibiting cell proliferation in mice with NMDA-induced retinal damage. However, when YAP is unrestrained through genetic manipulations, Müller cells undergo proliferation and differentiate into neuronal cells, entering a high-value state. This highlights the significance of the Hippo signaling pathway in Müller cell reprogramming (Hamon et al., 2019; Rueda et al., 2019).

Previous investigations have highlighted that retinal injury provokes the activation of pSmad3 signaling in Müller glia cells that respond to injury. However, in contrast to these findings, Lee’s study has revealed that pSmad3 expression is predominantly observed in quiescent Müller glia cells and is dampened in Müller glia cells responding to injury. Furthermore, their research has pinpointed TGF-β3 as the key ligand responsible for regulating pSmad3 expression. Intriguingly, only TGF-β3 exerts an inhibitory effect on injury-induced Müller glia proliferation, indicating the participation of a non-canonical TGF-β signaling pathway in this intricate process (Lee et al., 2020).

TGF-β has been shown to play a role in Müller cell reprogramming (Close et al., 2005), while Shh signaling has also been implicated in this process (Wan et al., 2007). Furthermore, Jak-STAT signaling has been identified as another pathway involved in the reprogramming of Müller cells (Peterson et al., 2000). Hoang’s studies demonstrated that the deletion of nuclear factor I factors a, b, and x (Nfia/b/x), which are responsible for preserving and reinstating a quiescent state in glia cells, led to the reprogramming of Müller glia into retinal bipolar and amacrine interneurons in adult mice following injury. These findings emphasize the intricate nature of the molecular mechanisms driving Müller cell reprogramming (Table 2).

**TABLE 2:** Signal pathways related Muller cell reprogramming.

Related molecules	Function	References
Lin28	With ASCL1, stimulating significantly Muller cells’ proliferative responses	Ueki et al., 2015
miR-5, miR-124 and miR-9	Overexpression of microRNAs to enhance the reprogramming effect of ASCL1	Wohl and Reh, 2016
Notch pathway	Enhance in chromatin accessibility and the expression of specific genes linked to regeneration in the uninjured retina	Sahu et al., 2021
	Depletion of Notch1a, Notch1b, Notch2 or Notch receptor ligands decreases cell proliferation during light damage	Campbell et al., 2021
	Suppressing the Notch pathway enhance photoreceptor	Fogerty et al., 2022
	Inhibition of the Notch pathway can induce the expression of ASCL1 in Muller cells	Loffler et al., 2015
	Treatment with the Notch signaling pathway ligand Jag1 promotes Muller re-entry into the cell cycle	Del Debbio et al., 2010
	Work synergistically with the Wnt pathway to promote the formation and proliferation of photoreceptors reprogrammed by Muller cells	Yao et al., 2016
Hippo pathway	A YAP-EGFR axis by which Müller cells exit their quiescence state	Hamon et al., 2019
Wnt pathway	βcatenin/Wnt/Lin28	Yao et al., 2016
TGF-β signaling	pSmad3 expression in quiescent Müller glia cells; TGF-β3 as the key ligand responsible for regulating pSmad3 expression and exerts an inhibitory effect on injury-induced Müller glia proliferation	Lee et al., 2020
	Maintains mitotic quiescence in the postnatal rat retina	Close et al., 2005
Shh signaling	Stimulate proliferation of Müller glia; enhances neurogenic potential by producing more rhodopsin-positive photoreceptors from Müller glia-derived cells	Wan et al., 2007
Jak-STAT signaling	STAT potentially directs Ascl1 to developmentally inappropriate targets	Jorstad et al., 2020

## Discussion

The capacity to regenerate retinal neurons following injury varies significantly across vertebrate species. Teleost fish possess the remarkable ability to regenerate all major retinal cell types after injury by reprogramming Müller glia. In the post-hatch chick, Müller glia can generate a limited number of neurons after injury, but this regenerative ability diminishes with age. Hoang’s research has presented a compelling discovery involving the identification of evolutionarily conserved and species-specific gene regulatory networks through a rigorous cross-species analysis. This network exerts precise control over the transitions of quiescent, reactive, and proliferative Müller glia in response to various stimuli. Notably, the distinctions in this regulatory network are instrumental in determining the diverse responses of Müller cells across different species when faced with injurious stimuli. In the context of mice, a dedicated network demonstrates its remarkable capacity to reinstate Müller glia to a quiescent state following injury. Conversely, in the cases of zebrafish and chick, the genes selectively expressed in reactive Müller glia actively promote a transition toward a proliferative and neurogenic progenitor state (Hoang et al., 2020). Indeed, in stark contrast to their counterparts in lower species, mammalian Müller cells display restricted and inefficient retinal repair capabilities. They cannot directly differentiate into retinal neurons to restore damaged areas and instead hinder the regenerative process through reactive gliosis. There has been debate regarding their neurogenic potential, as they do not function as such in the undamaged retina. Nevertheless, recent studies and advancements in understanding cell fate regulation have unveiled the immense stemness potential of Müller cells and provided avenues for harnessing it, such as through reprogramming. These discoveries hold great promise for advancing therapies targeting Müller cell-dependent retinal regeneration in mammals.

The concept of reprogramming has been a groundbreaking pursuit, starting with early cell fusion experiments (Brown and Fisher, 2021). Its culminated in the development of induced pluripotent stem cells (iPSCs) in 2006 (Takahashi and Yamanaka, 2006), opening a new era in reprogramming research. Since then, scientists have explored various transcription factors, compounds, and small molecules for their potential in reprogramming (Mahato et al., 2020; Pan et al., 2021). This exploration of cell fate regulation has deepened our understanding of the intricacies of cellular identity (Wang et al., 2021). The convergence of reprogramming research and the limited differentiation capacity of Müller cells in mammals has spurred the exploration and application of the neurogenic potential inherent in these cells.

Müller cells in mammals are typically “quiescent,” even when retinal damage occurs, microglia activation inhibits the reprogramming of Müller cells. Therefore, necessitating research into efficient ways to unlock their neurogenic potential. Comparative studies have highlighted variations in ASCL1 expression between species and in zebrafish Müller cells during reprogramming, underscoring the importance of this factor in the process. Indeed, overexpressing ASCL1 has proven effective in reprogramming mammalian Müller cells, and several pathways, including the ASCL1-Wnt signaling axis, ASCL1-Lin28-let7, Notch pathway, JAK/STAT, and HDAC inhibitors, have been identified to contribute to this process. Furthermore, introducing β-catenin and augmenting YAP, a core molecule in the Hippo pathway, have shown promise in achieving reprogramming. It is noteworthy that the investigation of these two molecules has revealed a complex interplay between the Hippo and Wnt pathways, featuring intricate context-dependent interactions of both positive and negative nature. This complexity underscores the need for a thorough understanding of the cellular processes and mechanisms involving Müller cells in response to β-catenin (Wang and Martin, 2017). This prompts questions regarding the initial identity of Müller glia expressing β-catenin prior to rod induction. Are these cells reprogrammed into a state resembling retinal progenitor cells (RPCs), thus acquiring the capacity for neurogenesis? Additionally, a puzzling observation arises in the context of the GFAP promoter, which remains active in β-catenin-expressing Müller glia, in contrast to their YAP5SA-reprogrammed counterparts (Heallen et al., 2011; Martin and Poché, 2019).

While the findings from studies on the knockdown of PTBP1, leading to the conversion of Müller glia into RGCs, have initially sparked considerable optimism and encouragement, subsequent investigations with uncertain outcomes regarding the efficacy of PTBP1 knockdown have urged us to approach these results with increased caution. This is especially pertinent when employing rigorous fate mapping techniques and lineage-tracing methods in the investigation of glia-to-neuron conversion within the retina. While the involvement of miRNAs in reprogramming is well-established, their specific effects on mammalian Müller cell reprogramming remain to be fully explored. Epigenetic modifications are also crucial in unraveling the stemness potential of mammalian Müller cells, and studies have demonstrated that demethylation promotes chromatin activation and epigenetic modifications, overcoming age-related limitations and enhancing reprogramming efficiency.

Despite the accumulation of substantial data in the past decades regarding the induced differentiation of mammalian Müller cells, many fundamental questions remain unanswered in this burgeoning field. The limited stemness of Müller cells in mammals and the reprogramming limitations of different Müller cell subtypes are key enquiries. Additionally, the influence of microenvironmental changes and the role of cells like microglia in the damaged intraretinal environment on reprogramming necessitate exploration. For example, Microglia cells and infiltrating immune cells may play roles in the repair process of Müller cells. These cells can respond to injury by migration, phagocytosis, and the release of factors that may influence Müller glia cells, thereby initiating or enhancing their reprogramming (Godwin et al., 2013; Fischer et al., 2014). In mice, it has been observed that microglia serve as inhibitors of Ascl1-mediated retinal regeneration, suggesting that the innate immune system imposes limitations on the regenerative response to injury in the retina (Todd et al., 2020). Besides, Müller glia cells in the central and peripheral regions of the primate and human retina exhibit distinct morphologies, structures, and functions (Bringmann et al., 2018), so different Müller glia cell subtypes with differences in reprogramming capabilities. In mammals, certain progenitor-associated transcription factors, such as CHX10 and PAX6, are expressed in a subset of Müller glia cells (Roesch et al., 2008). Significantly, a recent investigation led by Zhang Chunli’s research team has utilized lineage tracing mouse lines and multiple adeno-associated viruses (AAVs), initiating persistent inquiries about the assertion that Ptbp1 knockdown fails to initiate *trans-*differentiation of glial cells into neurons in mice (Hoang et al., 2022). Furthermore, this research has advanced our understanding of the high leakage issue in AAV-based systems for tracing Müller glia. For instance, Gao’s study introduced the AAV9-hGFAP-Cre-ΔWPRE system, which, when compared to the conventional AAV9-hGFAP-Cre-WPRE labeling system, demonstrates enhanced efficiency and specificity in Müller glia labeling (Gao et al., 2022).

Subsequently, Chen G and Wang LL conducted a meticulous discussion on *in vivo* astrocyte-to-neuron conversion, addressing aspects such as AAV toxicity, stringent lineage tracing, and experimental design (Chen, 2021; Wang and Zhang, 2022). The latter, utilizing lineage mapping and retrograde tracing techniques, underscored deficiencies in the original research’s lineage tracing. It also systematically ruled out the impact of virus titer on fluorescence leakage issues and expressed substantial skepticism regarding the assertion that “the Cre-loxP recombination may have created a higher barrier for cell conversion” This thorough examination raises significant doubts about the reliability of the original study. This kind of research has resulted in the development of a safer, more efficient, and highly specific labeling system for Müller cells, which offers a promising tool for *in vivo* tracing of cell fate (Gao et al., 2022). To sum up, addressing these questions is crucial for comprehending the transition to reprogramming in mammalian Müller cells and holds potential for future clinical applications.

Harnessing the regenerative potential of endogenous Müller cells can offer a promising avenue for treating untreatable retinal diseases. While clinical trials involving embryonic stem cell or induced pluripotent stem cell-derived retinal cell transplants are underway, several challenges persist, including the complex and time-consuming *in vitro* cell culture process, intricate surgical procedures, potential tumor formation, immune rejection, and ethical concerns associated with transplantation. In contrast, exploring the regenerative capacity of mammalian Müller glia cells presents an appealing alternative that circumvents the issues related to *in vitro* cell derivation and transplantation. Moreover, Müller glia cells are non-tumorigenic, making them an attractive candidate for *in vivo* retinal self-repair in humans. While we have to acknowledge that the reprogramming of mammalian Müller cells is still in its early stages, it is imperative to underscore the importance of continued exploration in this direction. Further basic experimental evidence and, eventually, clinical studies are essential steps forward. Overcoming challenges such as mammalian self-limitations, post-injury gliosis effects, and age-related decline in potential is crucial. Nonetheless, the reprogramming of mature Müller cells into diverse cell types opens exciting prospects and represents a promising avenue toward realizing the ultimate goal of retinal regeneration.

## Author contributions

Y-MG: Visualization, Writing—original draft, Writing—review and editing. XJ: Investigation, Writing—review and editing, Data curation, Supervision, Visualization. JM: Writing—review and editing, Investigation. JH: Methodology, Writing—review and editing, Visualization. X-FH: Conceptualization, Funding acquisition, Supervision, Writing—review and editing, Methodology. LY: Conceptualization, Funding acquisition, Supervision, Writing—original draft, Writing—review and editing.
